# Influence of intravenous iron therapy on mortality and cardiovascular events of patients on hemodialysis: A meta-analysis

**DOI:** 10.17305/bb.2025.12652

**Published:** 2025-08-03

**Authors:** Yan Chen, Dian Zhao, Chong Huang, Yanxia Chen, Weiping Tu, Chengyun Xu

**Affiliations:** 1 Department of Nephrology, The Second Affiliated Hospital of Nanchang University, Nanchang, China; 2 Department of Nephrology, Yongxiu County People’s Hospital, Jiujiang, China

**Keywords:** Intravenous iron, hemodialysis, mortality, cardiovascular events, meta-analysis

## Abstract

Intravenous (IV) iron is widely utilized to manage anemia in patients undergoing maintenance hemodialysis; however, its long-term safety remains uncertain. This meta-analysis aimed to evaluate the impact of IV iron on all-cause mortality and major adverse cardiovascular events (MACEs) within this population. We conducted a systematic search of PubMed, Embase, Cochrane Library, Web of Science, Wanfang, and CNKI up to March 2025 for randomized controlled trials (RCTs) that compared IV iron with placebo/usual care, oral iron, or varying doses of IV iron in adult hemodialysis patients. The primary outcomes assessed were all-cause mortality and MACEs. Data were synthesized using a random-effects model, and the quality of evidence was evaluated employing the GRADE approach. A total of fifteen RCTs involving 4257 patients were included in the analysis. Compared to placebo/usual care, IV iron did not significantly affect all-cause mortality (odds ratio [OR]: 1.36; 95% confidence interval [CI]: 0.60–3.09) or MACEs (OR: 0.81; 95% CI: 0.43–1.55), with a moderate level of evidence. Furthermore, IV iron demonstrated no significant differences in mortality (OR: 0.58; 95% CI: 0.18–1.90) or MACEs (OR: 2.47; 95% CI: 0.37–16.34) when compared to oral iron, although the quality of evidence in this comparison was very low. High-dose IV iron was associated with a reduced mortality rate compared to low-dose IV iron (OR: 0.81; 95% CI: 0.67–0.97), though this result was influenced by a single large study. In conclusion, IV iron does not appear to increase mortality or MACEs relative to placebo or oral iron. While high-dose IV iron may decrease mortality, the evidence remains limited, indicating a need for further research.

## Introduction

Iron deficiency, with or without anemia, is highly prevalent among patients receiving maintenance hemodialysis due to chronic blood loss during dialysis sessions, impaired gastrointestinal absorption, inflammation-induced hepcidin elevation, and inadequate dietary intake [1, 2]. Estimates suggest that up to 65%–75% of hemodialysis patients have iron deficiency, and iron deficiency anemia (IDA) affects approximately 30%–45% of this population [3, 4]. IDA is associated with a broad spectrum of adverse clinical outcomes, including reduced exercise capacity, impaired quality of life, resistance to erythropoiesis-stimulating agents (ESAs), and increased risk of hospitalization and mortality [5, 6]. Addressing iron deficiency is, therefore, a central component of anemia management in patients with end-stage renal disease (ESRD) on dialysis [7].

Intravenous (IV) iron therapy is currently recommended as the first-line approach for iron repletion in hemodialysis patients due to its superior efficacy and bioavailability compared to oral iron, especially in the context of inflammation and impaired gut absorption [8]. Clinical guidelines, including those from Kidney Disease: Improving Global Outcomes (KDIGO) and other nephrology societies, advocate for IV iron supplementation to maintain target hemoglobin levels and reduce ESA requirements [9, 10]. Several randomized controlled trials (RCTs) have demonstrated that IV iron effectively improves hemoglobin levels and iron indices and reduces the need for high ESA doses [11–13]. However, concerns remain regarding the safety of long-term or high-dose IV iron administration [14]. Potential adverse effects include oxidative stress, infection risk, vascular calcification, and cardiovascular complications [15, 16]. Some studies have raised the possibility that excessive iron exposure may contribute to endothelial dysfunction or promote atherosclerosis, thereby adversely influencing long-term prognosis in this vulnerable population [17, 18].

Despite the growing use of IV iron in routine dialysis care, the long-term impact of this intervention on survival and cardiovascular outcomes remains uncertain [19]. Most existing studies on this topic have been observational in nature and are susceptible to confounding by indication, selection bias, and inadequate adjustment for comorbid conditions and ESA dosing [20–23]. A few RCTs have explored the prognostic effects of IV iron therapy, but their findings are heterogeneous and often limited by small sample size, short follow-up duration, and variability of comparators [24–38]. Moreover, variation in iron formulation, dosing strategies, and baseline patient characteristics complicates the interpretation of results. Therefore, we conducted a comprehensive meta-analysis of RCTs to systematically evaluate the influence of IV iron therapy—compared with placebo/usual care, oral iron, and different IV iron dosing strategies—on all-cause mortality and major adverse cardiovascular events (MACEs) in adult patients undergoing maintenance hemodialysis.

## Materials and methods

During the design and implementation of this study, we followed the guidelines set forth by Preferred Reporting Items for Systematic Reviews and Meta-Analyses (PRISMA) [39, 40] and the Cochrane Handbook [41]. The protocol of this meta-analysis was prospectively registered in PROSPERO (registration number: CRD420251048379; https://www.crd.york.ac.uk/PROSPERO/view/CRD420251048379).

### Study inclusion and exclusion criteria

This meta-analysis aims to evaluate the effects of IV iron therapy on all-cause mortality and MACEs in adult patients with ESRD undergoing maintenance hemodialysis by comparing IV iron to placebo/usual care, oral iron, and different IV iron dosing strategies. The inclusion criteria were designated according to the aim of the meta-analysis and the PICOS principle.

P (Patients): Adult patients (aged ≥ 18 years) undergoing maintenance hemodialysis for ESRD.

I (Intervention): Intravenous (IV) iron therapy, including dialysate-administered iron (e.g., ferric pyrophosphate citrate [FPC]).

C (Comparators): According to the aim of the meta-analysis, comparators included (1) placebo or usual care (no iron supplementation); (2) oral iron therapy; and (3) lower-dose IV iron (for high vs low IV iron dose comparison).

O (Outcome): Incidence of all-cause mortality and MACEs compared between hemodialysis patients who were allocated to the IV iron and control groups. In general, MACEs were defined as a composite outcome of myocardial infarction, stroke, heart failure, and cardiovascular death.

S (Study design): RCTs with a parallel design published as full-text articles in English or Chinese.

Excluded from the analysis were reviews, editorials, meta-analyses, studies not designed as RCTs, studies involving pediatric patients, patients not on hemodialysis, not receiving IV iron, or not reporting the outcomes of interest. To minimize the risk of overlapping patient populations, we carefully compared study characteristics—including author teams, study centers, recruitment periods, and sample sizes. When overlap was suspected, the study with the larger and more complete dataset was included, and the duplicate was excluded. This resulted in the exclusion of one study due to overlapping data.

### Database search

The Medline (PubMed), Embase (Ovid), CENTER (Cochrane Library), Web of Science, Wanfang, and China National Knowledge Infrastructure (CNKI) databases were searched using the combination of the following terms: (1) “iron repletion” OR “intravenous iron” OR “ferric carboxymaltose” OR “ferric derisomaltose” OR “iron isomaltoside 1000” OR “iron sucrose” OR “iron supplementation” OR “iron therapy” OR “Ferumoxytol” OR “ferric pyrophosphate citrate”; (2) “dialysis” OR “hemodialysis”; (3) “random” OR “randomized” OR “randomised” OR “randomly” OR “control” OR “placebo”; and (4) “mortality” OR “death” OR “adverse events” OR “heart” OR “cardiac” OR “deaths” OR “survival” OR “cardiovascular” OR “prognosis,” limited to clinical studies in humans. Only studies that included human subjects and were published in English or Chinese were considered. The full search strategy for each database is shown in Supplemental data. Additionally, references to related reviews and original articles were screened as part of the final database search. The final database search was conducted on March 30, 2025.

### Data collection and quality evaluation

Two authors conducted independent database searches, data collection, and quality assessments. In the event of disagreements, discussions were held with the corresponding author. The data collected encompassed various aspects, including overall study information (such as the first author and publication year), study design (double-blind, single-blind, or open-label), participant characteristics (diagnosis of the patients, sample size, mean ages, and sex distribution), details of interventions and controls, follow-up durations, and outcomes reported. The quality of the included RCTs was assessed using the Cochrane Risk of Bias Tool [41]. This tool evaluated various aspects such as random-sequence generation, allocation concealment, blinding of participants and outcome assessment, addressing incomplete outcome data, selective reporting, and other sources of bias. In addition, two reviewers evaluated the certainty of evidence using the Grading of Recommendations, Assessment, Development, and Evaluation (GRADE) system, which includes risk of bias, inconsistency, indirectness, imprecision, and publication bias [42]. The certainty of evidence was classified as very low, low, moderate, or high. Disagreements were resolved by discussion with the corresponding author.

### Statistical analysis

The incidence of all-cause mortality and MACEs, compared between hemodialysis patients with IV iron vs placebo/usual care, IV iron vs oral iron, and high-dose vs low-dose IV iron, was summarized as odds ratios (OR) and corresponding 95% confidence intervals (CIs). Heterogeneity was assessed using the Cochrane *Q* test [41]. The *I*^2^ statistic was also calculated, with *I*^2^ < 25%, within 25%–75%, and > 75% indicating mild, moderate, and substantial heterogeneity [43]. A random-effects model was used to pool the results because this model could incorporate the potential influence of heterogeneity [41]. A sensitivity analysis by excluding one dataset at a time was performed to evaluate the robustness of the findings [41]. An evaluation of publication bias was conducted via a visual inspection using funnel plots and by performing Egger’s regression asymmetry test [44]. A *P* < 0.05 was considered statistically significant. Statistical analyses were conducted using RevMan (version 5.1; Cochrane, Oxford, UK) and Stata software (version 17.0; Stata Corporation, College Station, TX, USA).

## Results

### Literature search

**Figure 1.**
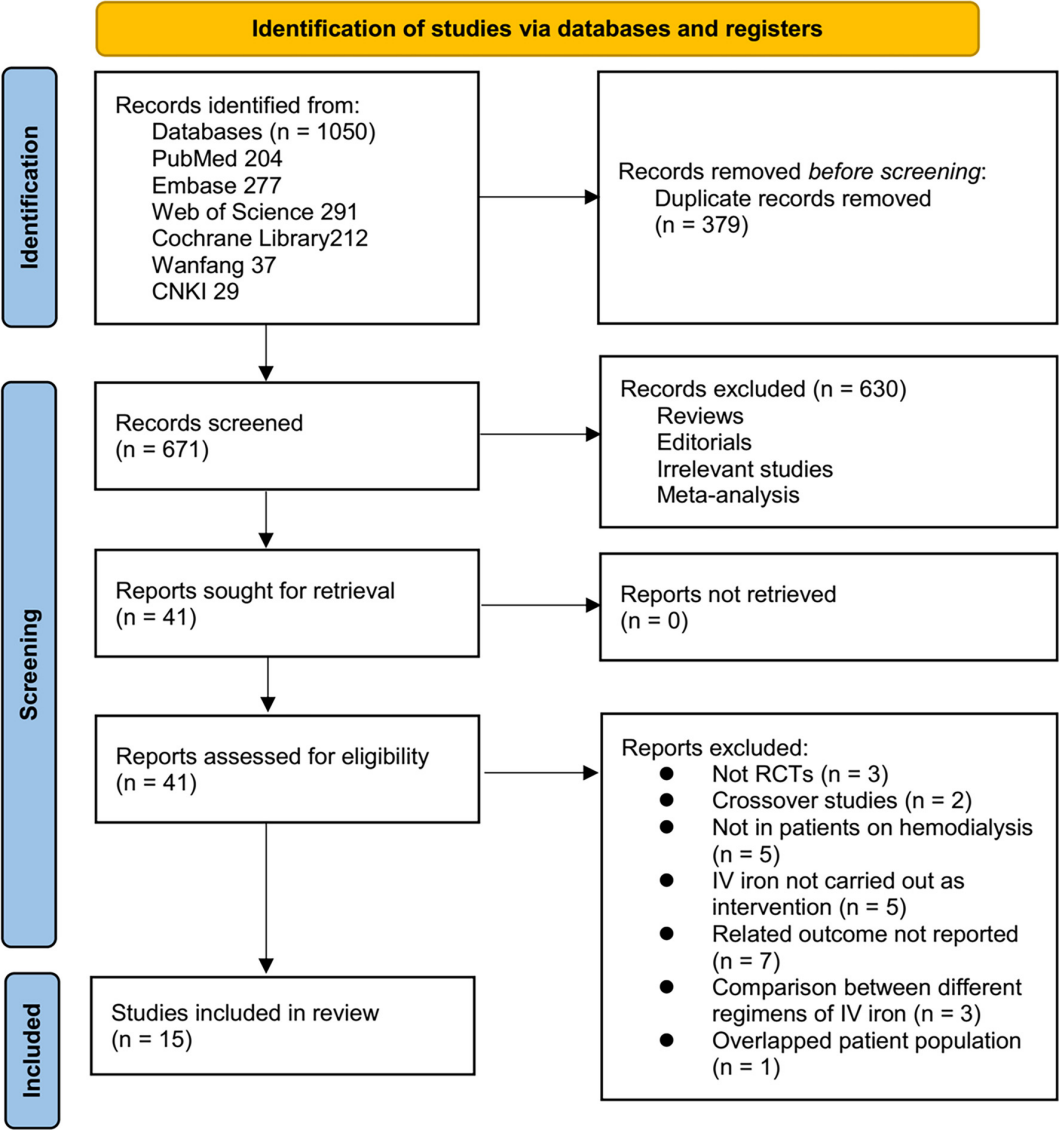
Flowchart for the literature search and study inclusion.

Figure 1 depicts the flowchart that outlines the process of database searching and study identification, ultimately leading to the selection of studies for inclusion. Initially, a total of 1050 articles were obtained through the database search, which was subsequently reduced to 671 after eliminating 379 duplicate records. Subsequently, 630 articles were excluded based on an evaluation of their titles and abstracts, primarily due to their lack of relevance to the objective of the present meta-analysis. Then, 26 out of the remaining 41 studies were excluded after full-text reviews for reasons outlined in Figure 1. Ultimately, 15 studies were included in the meta-analysis [24–38].

### Study characteristics

A total of 15 RCTs [24–38] were included in this meta-analysis, encompassing 4257 adult patients undergoing maintenance hemodialysis. Study characteristics are summarized in Table 1. Because one study [25] involved three interventional arms, including IV iron, oral iron, and usual care without iron supplementation, this study was included in two comparisons: IV iron vs placebo/usual care and IV iron vs oral iron. Among the included trials, five compared IV iron therapy to placebo or usual care without iron supplementation [25, 27, 28, 30, 31], four compared IV iron to oral iron [24, 25, 29, 38], and seven evaluated different IV iron dosing strategies (high vs low dose) [26, 32–37]. All studies were conducted in patients undergoing chronic hemodialysis, and all participants were adults (mean age range: 49.6–70.8 years). The proportion of male participants ranged from 44.5% to 69.0%.

**Table 1:** Characteristics of the included RCTs

**Study**	**Country**	**Design**	**Diagnosis**	**Patient number**	**Mean age (years)**	**Men (%)**	**Intervention**	**Control**	**Follow-up duration (months)**	**Events reported**
*IV iron vs placebo/usual care*										
Fudin, 1998	Israel	R, OL	Adults on maintenance HD with IDA	29	57.1	65.5	Intravenous sodium iron gluconate 62.5 mg weekly during dialysis (adjusted to 62.5–125 mg/month once transferrin saturation reached ∼35%)	Usual care without iron supplementation	22	All-cause death and MACEs
Kapoian, 2008	USA	R, OL	Anemia in adult hemodialysis patients with high ferritin (500–1200 ng/mL) and TSAT <25%, receiving adequate epoetin doses	112	59.8	49.1	Ferric gluconate 125 mg IV over 8 consecutive dialysis sessions (total 1 g)	Usual care without iron supplementation	3	All-cause death and MACEs
Kuo, 2018	Taiwan	R, SB	Adult chronic HD patients	110	59.5	49.5	IV iron sucrose 100 mg weekly for 12 weeks (total 1.2 g)	Normal saline (Placebo)	3	MACEs
Gupta, 2015	USA	R, DB	Adults on maintenance HD	103	59	61.2	FPC added to dialysate (2 µmol/L iron) at every dialysis session for 9 months	Standard dialysate (Placebo)	9	All-cause death and MACEs
Fishbane, 2015	USA and Canada	R, SB	Adults on maintenance HD	588	58.4	63.6	FPC via dialysate at each HD session (2 µmol/L iron) for up to 48 weeks	Standard dialysate (Placebo)	48	All-cause death
*IV iron vs PO iron*										
Fishbane, 1995	USA	R, OL	Hemodialysis patients with stable anemia and replete iron indices (ferritin >100 ng/mL, TSAT >15%)	52	49.6	62	IV iron dextran 100 mg twice weekly for 4 months during dialysis (total 3.2 g)	Oral iron (ferrous sulfate 325 mg TID or polysaccharide 150 mg BID)	4	All-cause death
Fudin, 1998	Israel	R, OL	Adults on maintenance HD with IDA on stable ESA	30	52.7	59	Intravenous sodium iron gluconate 62.5 mg weekly during dialysis (adjusted to 62.5–125 mg/month once transferrin saturation reached ∼35%)	Oral iron (ferrous sulfate 160 mg/day, i.e., ∼50 mg elemental iron in slow-release form)	26	All-cause death and MACEs
Provenzano, 2009	USA	R, OL	Anemic adult patients on HD receiving stable ESA therapy	230	60.2	56.6	IV ferumoxytol 510 mg × 2 doses within 7 days (total 1.02 g)	Oral iron (Ferro-Sequels) 200 mg elemental iron daily for 21 days	1	All-cause death
Lu, 2025	China	R, OL	Adults with anemia on maintenance HD, TSAT 20%–50%, ferritin 100–500 ng/mL	193	55.3	62.2	IV iron sucrose 100 mg biweekly for 24 weeks	Oral polysaccharide-iron complex 150 mg twice daily for 24 weeks	6	All-cause death and MACEs
*High-dose vs low dose IV iron*										
Besarab, 2000	USA	R, OL	Adults on chronic HD with stable hemoglobin (9.5–12 g/dL) and TSAT 19%–30%, ferritin 150–600 ng/mL	42	60.8	61.9	Loading: 4–6 doses of IV iron dextran (100 mg each over 2 weeks) and maintenance: 25–150 mg weekly to maintain TSAT between 30%--50%	IV iron dextran 25–150 mg weekly to maintain TSAT between 20%--30% for 6 months	6	All-cause death and MACEs
Macdougall, 2019	UK	R, OL	Adults on maintenance hemodialysis for ≤12 months, with ferritin <400 µg/L and TSAT <30%, all receiving ESAs	2141	62.8	65.3	Proactive IV iron sucrose 400 mg/month unless ferritin >700 µg/L or TSAT ≥40%	Reactive IV iron sucrose 0–400 mg/month, triggered when ferritin <200 µg/L or TSAT <20%	25	All-cause death and MACEs
Susantitaphong, 2020	Thailand	R, OL	Chronic HD patients with functional IDA (TSAT <30%, ferritin 200–400 ng/mL)	200	52.8	53.5	IV iron to maintain ferritin 600–700 ng/mL (∼192 mg/month); initial loading dose of 600 mg over 6 weeks	IV iron to maintain ferritin 200–400 ng/mL (∼108 mg/month); no loading dose	6	All-cause death and MACEs
van den Oever, 2020	The Netherlands	R, SB	Maintenance HD patients treated with darbepoetin alfa	200	68.9	69	Pharmacist-guided algorithm with iron sucrose, typically 100 mg per dose (median 75 mg/week), administered once weekly or more frequently (up to three times per week), depending on iron status	Iron sucrose use at the discretion of the nephrologist; median dose was 0 mg/week	11	All-cause death
Zununi Vahed, 2021	Iran	R, DB	HD patients with ferritin <700 ng/mL or TSAT <40%, on stable dialysis	60	61.2	59.6	IV iron 400 mg/month (100 mg/week) for 6 months unless ferritin >700 ng/mL or TSAT ≥40%	IV iron 100 mg/week only if ferritin <200 ng/mL or TSAT <20%	6	MACEs
Gu, 2023	China	R, OL	Maintenance HD patients with TSAT 20–50%, ferritin 200–500 µg/L, Hb <130 g/L, all receiving rHuEPO	80	58.9	56.2	Iron sucrose 100 mg weekly	Iron sucrose 100 mg every two weeks	36	All-cause death
Anumas, 2023	Thailand	R, DB	Chronic HD patients with TSAT 20%–40%, ferritin 200–700 ng/mL, and on ESA	79	70.8	44.5	IV iron sucrose 200 mg/month (as 100 mg every 2 weeks)	IV iron sucrose 100 mg/month (single monthly dose)	12	All-cause death and MACEs

Abbreviations: R: Randomized; OL: Open-label; SB: Single-blind; DB: Double-blind; HD: Hemodialysis; IDA: Iron deficiency anemia; TSAT: Transferrin saturation; ESA: Erythropoiesis-stimulating agent; FPC: Ferric pyrophosphate citrate; IV: Intravenous; PO: Per os (oral); MACEs: Major adverse cardiovascular events; Hb: Hemoglobin; rHuEPO: Recombinant human erythropoietin; TID: Three times daily; BID: Twice daily; RCT: Randomized controlled trial.

In brief, IV iron vs placebo/usual care was evaluated in five trials from Israel, the USA, Taiwan, and Canada [25, 27, 28, 30, 31]. These studies included a range of IV iron formulations: sodium ferric gluconate [25, 27], iron sucrose [28], and FPC administered via dialysate [30, 31]. Sample sizes ranged from 29–588 patients. Follow-up durations varied from 3–48 months. The outcomes of all-cause mortality [25, 27, 28, 30] and MACEs [25, 27, 28, 31] were reported in four studies separately.

IV iron vs oral iron was examined in four open-label RCTs conducted in the USA, Israel, and China [24, 25, 29, 38]. IV iron regimens included iron dextran [24], sodium ferric gluconate [25], ferumoxytol [29], and iron sucrose [38]. Oral comparators included ferrous sulfate, Ferro-Sequels, or polysaccharide-iron complexes, with elemental iron doses ranging from 50–200 mg/day. Sample sizes ranged from 30 to 230 patients, and follow-up durations ranged from 1 to 6 months. All studies reported mortality outcomes, while two studies also included MACEs [25, 38].

High-dose vs low-dose IV iron was analyzed in seven trials from various countries, including the USA, UK, Thailand, The Netherlands, Iran, and China [26, 32–37]. Iron formulations included iron dextran [26] and iron sucrose [32–37], compared between a high-dose and a low-dose group. Follow-up durations ranged from 6 to 36 months. Six studies reported mortality outcomes [26, 32–34, 36, 37], and five studies included data on MACEs [26, 32, 33, 35, 36].

### Study quality evaluation

Details of the risk of bias assessment are provided in Table 2. Fourteen studies were judged to have low risk in random sequence generation [25–38]. Nine studies [28–32, 34–36, 38] adequately reported the details of allocation concealment. Blinding of participants was judged to be adequate in six studies [28, 30, 31, 34–36], while blinding of outcome assessment was considered to be adequate in four studies [31–33, 36]. Despite these limitations, all studies addressed incomplete outcome data appropriately and had no evidence of selective reporting or other major sources of bias.

**Table 2:** Study quality evaluation via the Cochrane Risk of Bias Tool

**Study**	**Random sequence generation**	**Allocation concealment**	**Blinding of participants**	**Blinding of outcome assessment**	**Incomplete outcome data addressed**	**Selective reporting**	**Other sources of bias**
*IV iron vs placebo/usual care*							
Fudin, 1998	Low risk	Unclear	High risk	Unclear	Low risk	Low risk	Low risk
Kapoian, 2008	Low risk	Low risk	High risk	Unclear	Low risk	Low risk	Low risk
Kuo, 2018	Low risk	Unclear	Low risk	Unclear	Low risk	Low risk	Low risk
Gupta, 2015	Low risk	Low risk	Low risk	Low risk	Low risk	Low risk	Low risk
Fishbane, 2015	Low risk	Low risk	Low risk	Unclear	Low risk	Low risk	Low risk
*IV iron vs PO iron*							
Fishbane, 1995	Unclear	Unclear	High risk	Unclear	Low risk	Low risk	Low risk
Fudin, 1998	Low risk	Unclear	High risk	Unclear	Low risk	Low risk	Low risk
Provenzano, 2009	Low risk	Low risk	High risk	Unclear	Low risk	Low risk	Low risk
Lu, 2025	Low risk	Low risk	High risk	Unclear	Low risk	Low risk	Low risk
*High-dose vs low dose IV iron*							
Besarab, 2000	Low risk	Unclear	High risk	Unclear	Low risk	Low risk	Low risk
Macdougall, 2019	Low risk	Low risk	High risk	Low risk	Low risk	Low risk	Low risk
Susantitaphong, 2020	Low risk	Unclear	High risk	Low risk	Low risk	Low risk	Low risk
van den Oever, 2020	Low risk	Low risk	Low risk	Unclear	Low risk	Low risk	Low risk
Zununi Vahed, 2021	Low risk	Low risk	Low risk	Unclear	Low risk	Low risk	Low risk
Gu, 2023	Low risk	Unclear	High risk	Unclear	Low risk	Low risk	Low risk
Anumas, 2023	Low risk	Low risk	Low risk	Low risk	Low risk	Low risk	Low risk

Abbreviation: IV: Intravenous.

**Figure 2.**
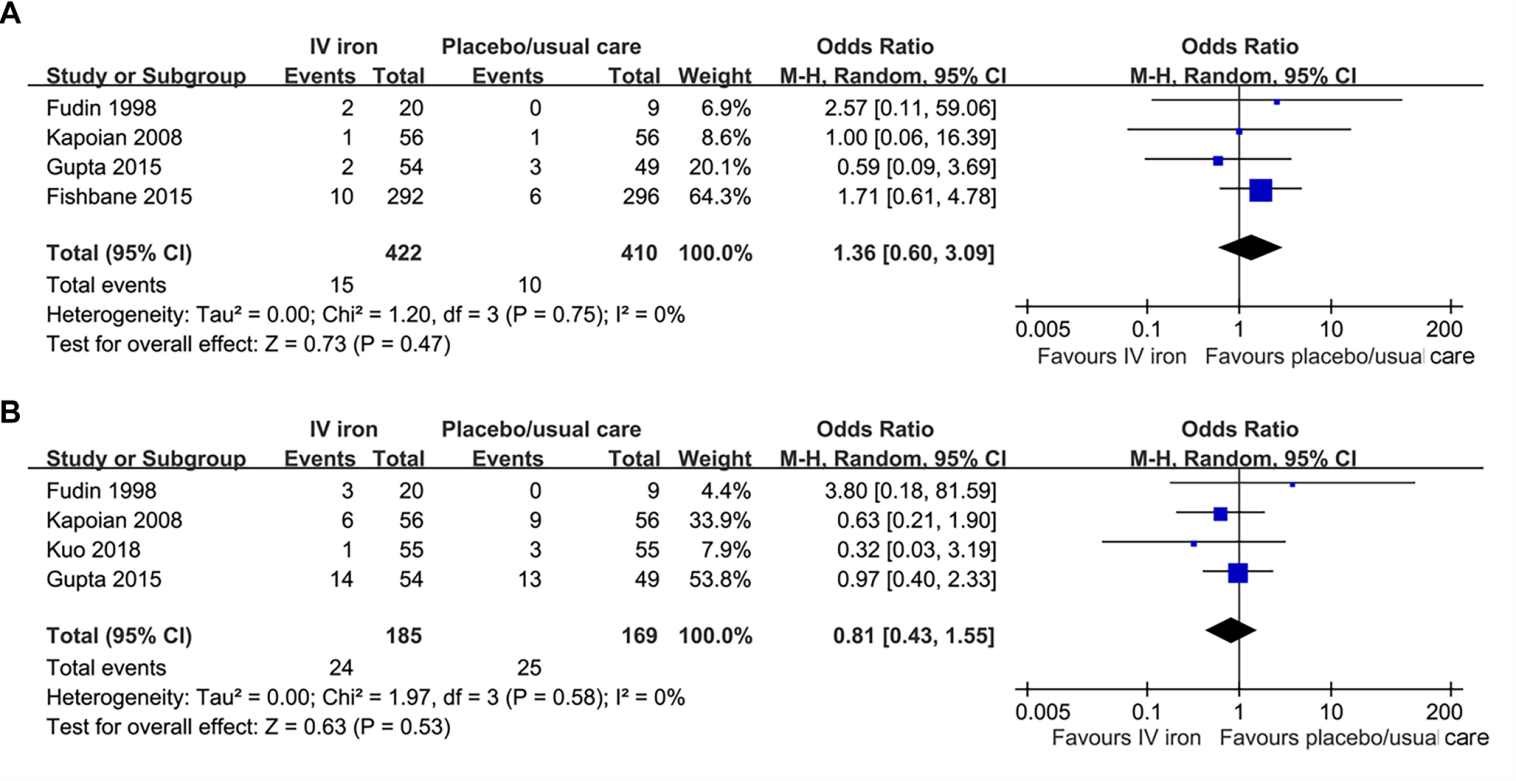
**Forest plots for the meta-analyses comparing the influences of IV iron** **vs** **placebo/usual care on the risk of all-cause mortality and MACEs in patients on hemodialysis.** (A) Forest plots for the meta-analysis of all-cause mortality; (B) Forest plots for the meta-analysis of MACEs. Abbreviations: IV: Intravenous; MACE: Major adverse cardiovascular event.

### IV iron vs placebo/usual care

The pooled results of four studies showed that compared to placebo/usual care, IV iron did not significantly affect the risk of all-cause mortality [25, 27, 28, 30] (OR: 1.36, 95% CI: 0.60–3.09, *P* ═ 0.47; Figure 2A) or MACEs [25, 27, 28, 31] (OR: 0.81, 95% CI: 0.43–1.55, *P* ═ 0.53; Figure 2B) in patients on hemodialysis, with no significant heterogeneity (both *I*^2^ ═ 0%). The summarized certainty of evidence using the GRADE system is shown in Table 3. The certainty of the evidence was downgraded one level due to suspected publication bias arising from the limited number of included studies. As a result, the overall certainty was judged to be moderate. Sensitivity analyses excluding one study at a time yielded consistent results (OR: 0.89–1.67 for all-cause mortality; OR: 0.66–0.93 for MACEs; *P* all > 0.05).

### IV iron vs oral iron

**Figure 3.**
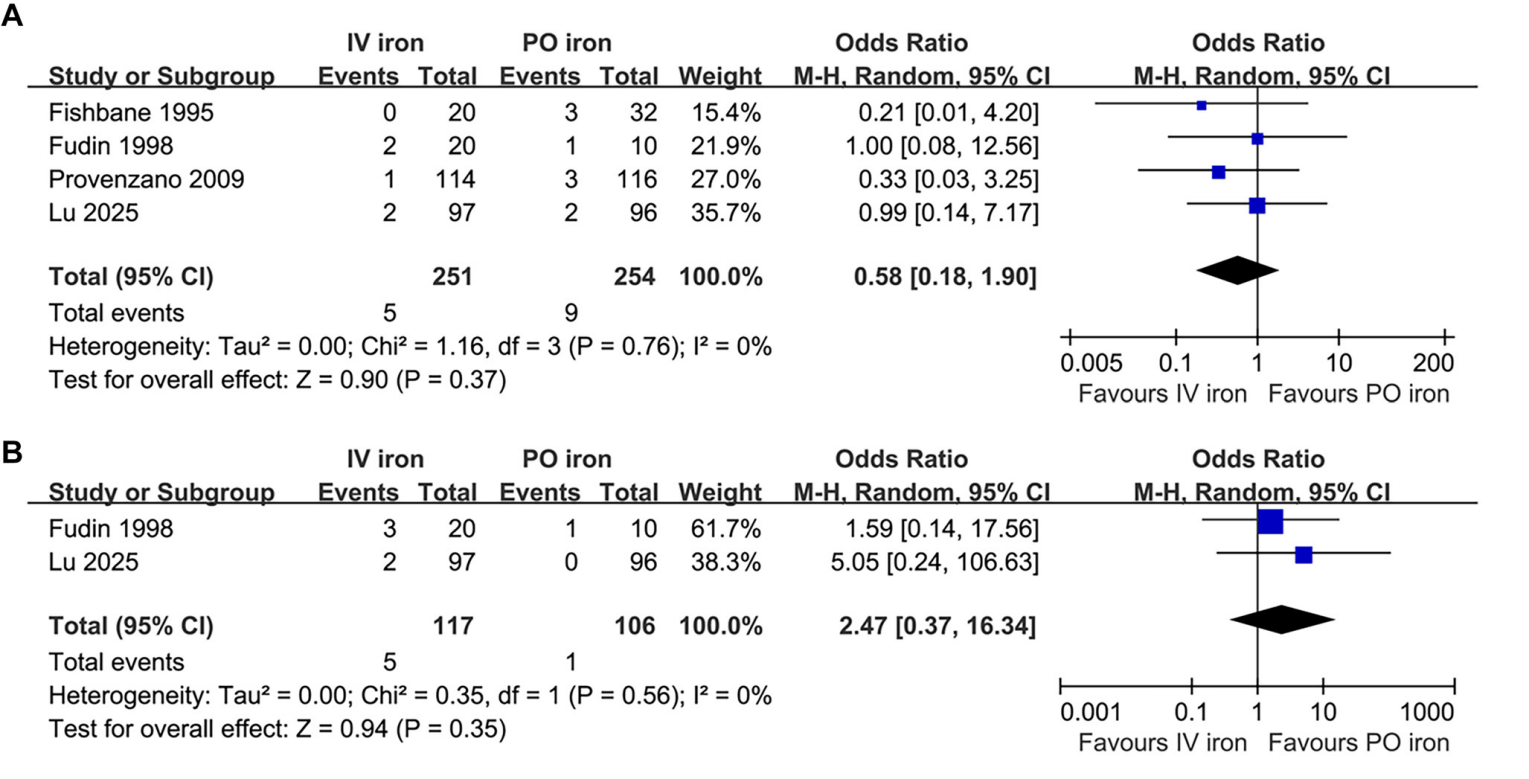
**Forest plots for the meta-analyses comparing the influences of IV iron** **vs** **oral iron on the risk of all-cause mortality and MACEs in patients on hemodialysis.** (A) Forest plots for the meta-analysis of all-cause mortality; (B) Forest plots for the meta-analysis of MACEs. Abbreviations: IV: Intravenous; MACE: Major adverse cardiovascular event.

Further meta-analysis of four studies [24, 25, 29, 38] suggested that IV iron did not significantly affect the risk of all-cause mortality compared to oral iron (OR: 0.58, 95% CI: 0.18–1.90, *P* ═ 0.37; Figure 3A) with no significant heterogeneity (*I*^2^ ═ 0%). The sensitivity analyses by excluding one study at a time did not significantly affect the finding (OR: 0.43–0.71, *P* all > 0.05). In addition, the pooled results of two studies [25, 38] showed that IV iron demonstrated no significant difference in MACE incidence compared to oral iron (OR: 2.47, 95% CI: 0.37–16.34, *P* ═ 0.35; Figure 3A) with no significant heterogeneity (*I*^2^ ═ 0%). The certainty of the evidence was downgraded by three levels to very low. This was due to the open-label design of all included studies, imprecision arising from wide CIs caused by low event rates, and potential publication bias related to the small number of available studies (Table 3).

### High-dose vs low-dose IV iron

**Figure 4.**
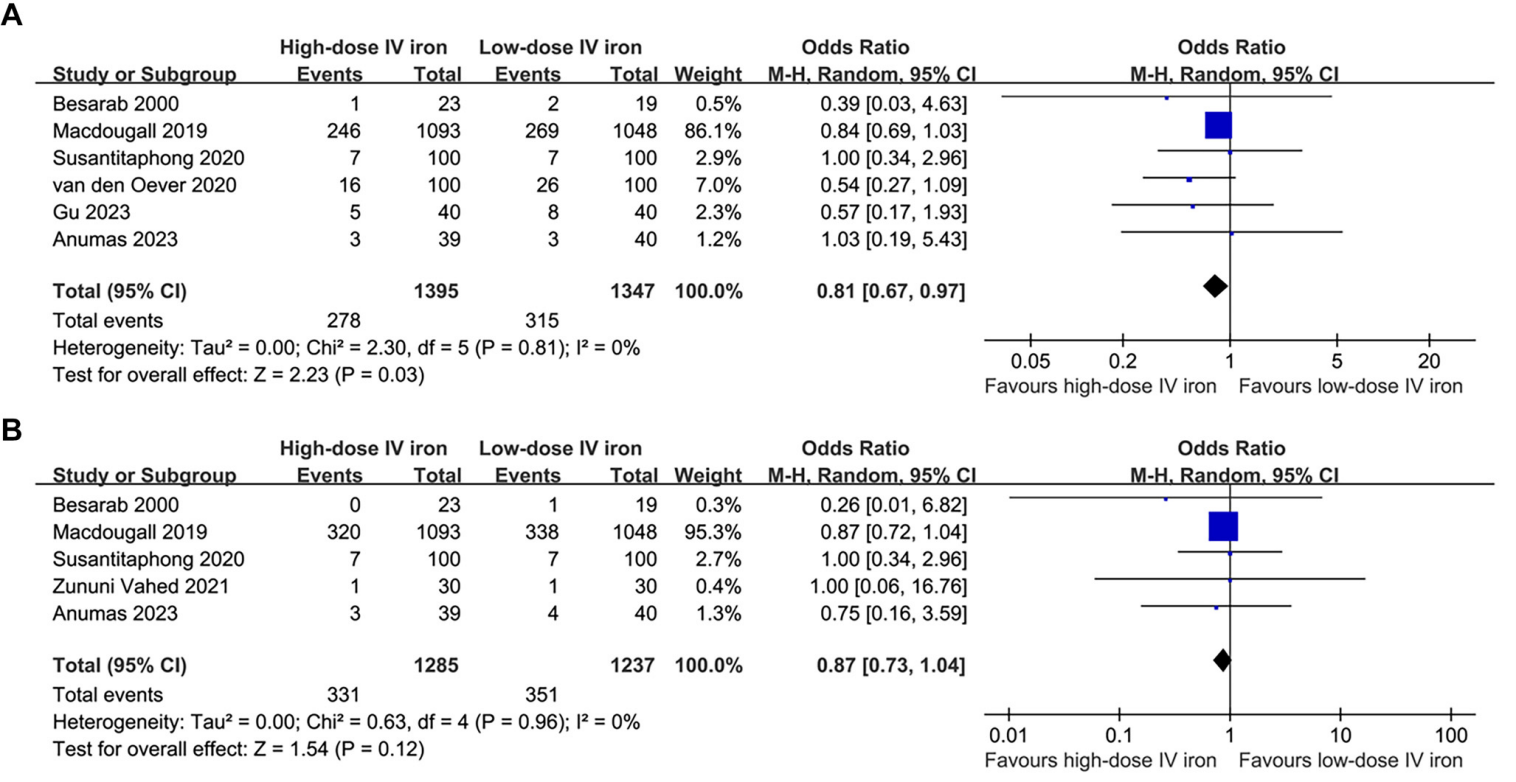
**Forest plots for the meta-analyses comparing the influences of high-dose IV iron** **vs** **low-dose IV iron on the risk of all-cause mortality and MACEs in patients on hemodialysis.** (A) Forest plots for the meta-analysis of all-cause mortality; (B) Forest plots for the meta-analysis of MACEs. Abbreviations: IV: Intravenous; MACE: Major adverse cardiovascular event.

The pooled results of six studies [26, 32–34, 36, 37] suggested that high-dose IV iron could significantly reduce the risk of all-cause mortality in these patients compared to low-dose IV iron (OR: 0.81, 95% CI: 0.67–0.97, *P* ═ 0.03; Figure 4A) with no significant heterogeneity (*I*^2^ ═ 0%). However, sensitivity analysis showed that the result becomes non-significant after excluding the large-scale study by Macdougall et al. (OR: 0.65, 95% CI: 0.40–1.06, *P* ═ 0.08; *I*^2^ ═ 0%). Further meta-analysis including five RCTs [26, 32, 33, 35, 36] showed that high-dose IV iron did not significantly reduce MACEs compared to low-dose IV iron (OR: 0.87, 95% CI: 0.73–1.04, *P* ═ 0.12; *I*^2^ ═ 0%; Figure 4B). The sensitivity analyses by excluding one study at a time did not significantly affect the finding (OR: 0.85–0.87, *P* all > 0.05). The certainty of the evidence was downgraded by one level to moderate due to potential publication bias stemming from the limited number of included studies (Table 3).

### Publication bias

**Figure 5.**
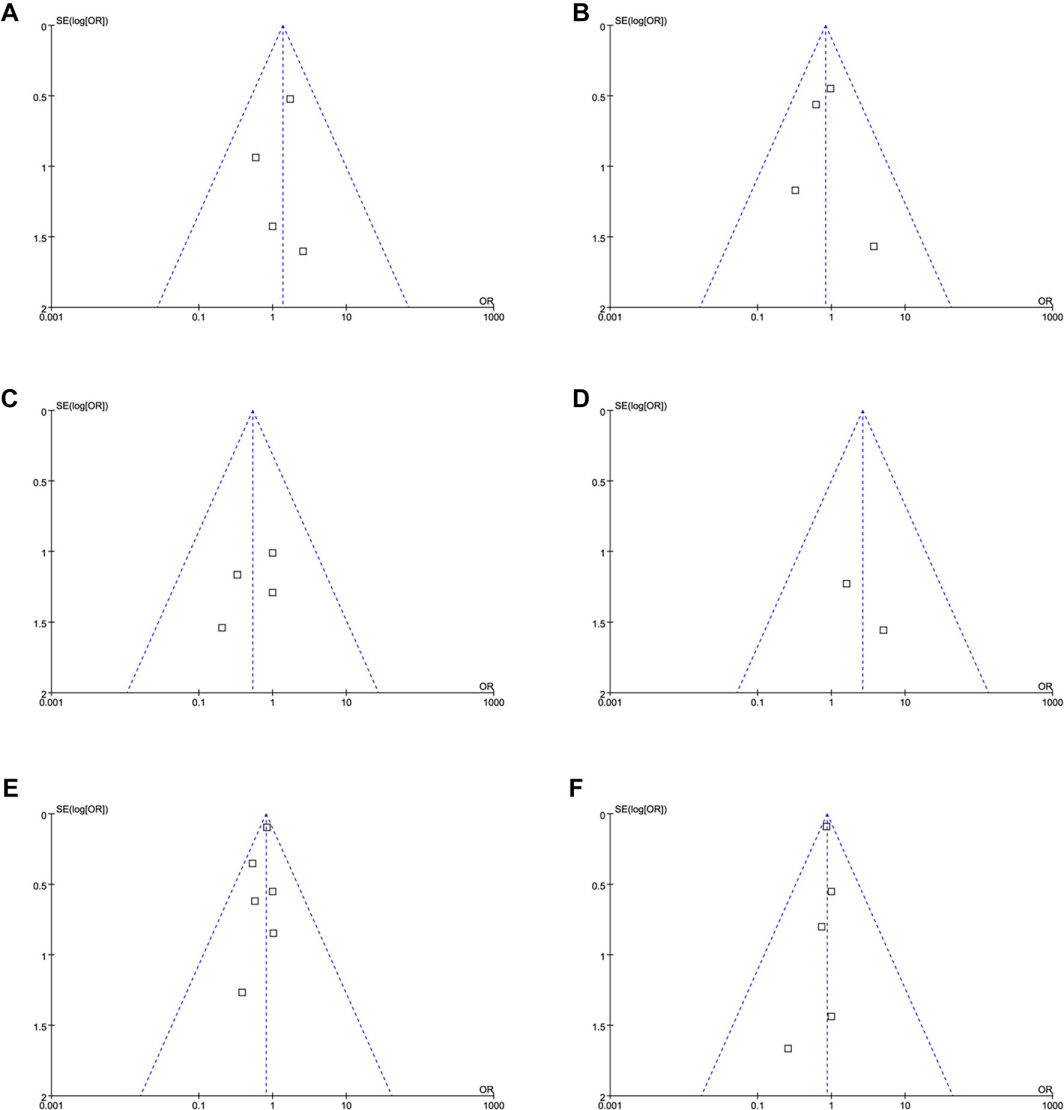
**Funnel plots evaluating the publication bias underlying the meta-analyses.** (A) Funnel plots for the meta-analysis comparing IV iron vs placebo/usual care on all-cause mortality; (B) Funnel plots for the meta-analysis comparing IV iron vs placebo/usual care on MACEs; (C) Funnel plots for the meta-analysis comparing IV iron vs oral iron on all-cause mortality; (D) Funnel plots for the meta-analysis comparing IV iron vs oral iron on MACEs; (E) Funnel plots for the meta-analysis comparing high-dose IV iron vs low-dose IV iron on all-cause mortality; and (F) Funnel plots for the meta-analysis comparing high-dose IV iron vs low-dose IV iron on MACEs. Abbreviations: IV: Intravenous; MACE: Major adverse cardiovascular event.

Funnel plots for the meta-analyses comparing all-cause mortality and MACEs across IV iron vs placebo/usual care, IV iron vs oral iron, and high-dose vs low-dose IV iron are presented in Figure 5A–5F. Although these plots appear roughly symmetrical on visual inspection, the small number of studies included for each outcome (ranging from 2 to 6) limits the reliability of this assessment. As a result, publication bias cannot be excluded, and Egger’s regression tests were not performed due to insufficient statistical power.

## Discussion

In this meta-analysis of 15 RCTs including 4257 adult patients undergoing maintenance hemodialysis, we systematically evaluated the impact of IV iron therapy on all-cause mortality and MACEs. We assessed three key clinical comparisons: IV iron vs placebo/usual care, IV iron vs oral iron, and high-dose vs low-dose IV iron. Overall, our findings suggest that IV iron therapy does not significantly increase the risk of mortality or cardiovascular events when compared with control interventions. Moreover, high-dose IV iron may offer a survival benefit compared to lower-dose regimens, although this finding should be interpreted cautiously due to limitations in the underlying evidence.

**Table 3:** Summarized certainty of evidence using the GRADE system

**Outcome**	**Quality assessment**							**Absolute effect** **OR (95% CI)**	**Quality**
	**No. of studies**	**Design**	**Risk of bias**	**Inconsistency**	**Indirectness**	**Imprecision**	**Other considerations**		
OR for the risk of all-cause mortality of HD patients receiving IV iron vs placebo/usual care	4	RCTs	No serious risk of bias	No serious inconsistency	No serious indirectness	No serious imprecision	Possible publication bias due to limited number of studies included	1.36 (0.60 to 3.09)	⊕ ⊕ ⊕O MODERATE
OR for the risk of MACEs of HD patients receiving IV iron vs placebo/usual care	4	RCTs	No serious risk of bias	No serious inconsistency	No serious indirectness	No serious imprecision	Possible publication bias due to limited number of studies included	0.81 (0.43 to 1.55)	⊕ ⊕ ⊕O MODERATE
OR for the risk of all-cause mortality of HD patients receiving IV iron vs PO iron	4	RCTs	Serious risk of bias (all OL RCTs)	No serious inconsistency	No serious indirectness	Serious imprecision (wide CI due to low events)	Possible publication bias due to limited number of studies included	0.58 (0.18 to 1.90)	⊕OOO VERY LOW
OR for the risk of MACEs of HD patients receiving IV iron vs PO iron	2	RCTs	Serious risk of bias (all OL RCTs)	No serious inconsistency	No serious indirectness	Serious imprecision (wide CI due to low events)	Possible publication bias due to limited number of studies included	2.47 (0.37 to 16.34)	⊕OOO VERY LOW
OR for the risk of all-cause mortality of HD patients receiving high-dose vs low-dose IV iron	6	RCTs	No serious risk of bias	No serious inconsistency	No serious indirectness	No serious imprecision	Possible publication bias due to limited number of studies included	0.81 (0.67 to 0.97)	⊕ ⊕ ⊕O MODERATE
OR for the risk of all-cause mortality of HD patients receiving high-dose vs low-dose IV iron	5	RCTs	No serious risk of bias	No serious inconsistency	No serious indirectness	No serious imprecision	Possible publication bias due to limited number of studies included	0.87 (0.73 to 1.04)	⊕ ⊕ ⊕O MODERATE

Abbreviations: OR: Odds ratio; CI: Confidence interval; HD: Hemodialysis; IV: Intravenous; PO: Per os (oral); MACEs: Major adverse cardiovascular events; RCTs: Randomized controlled trials; OL: Open-label; GRADE: Grading of Recommendations, Assessment, Development and Evaluation. Specific reasons for each GRADE domain, including: Risk of bias: Downgraded if a significant proportion of studies had unclear or high risk of bias in key domains (e.g., random sequence generation, allocation concealment, or selective reporting). Inconsistency: Downgraded if substantial heterogeneity was observed (*I*^2^ > 50%) and could not be explained by subgroup analyses or meta-regression. Indirectness: Evaluated but not downgraded, as all included studies directly assessed the population and outcomes of interest. Imprecision: Downgraded if confidence intervals were wide, overlapping no effect, or if the overall sample size was small. Publication bias: Assessed using funnel plots and Egger’s test; downgraded if significant asymmetry suggested potential bias.

In the comparison of IV iron vs placebo or usual care without iron supplementation, the pooled results from four trials showed no significant association with all-cause mortality or MACEs. These findings are consistent with previous meta-analyses, such as the 2018 systematic review by Hougen et al. [45], which reported no increase in mortality, infection, or cardiovascular risk associated with higher IV iron doses in dialysis patients. Our analysis adds to this evidence by focusing exclusively on RCTs. The moderate certainty of evidence, however, reflects potential publication bias due to the small number of trials and relatively low event rates, limiting the statistical power to detect rare but clinically important adverse effects.

For the comparison of IV iron vs oral iron, the results similarly showed no significant differences in all-cause mortality or MACEs. However, the evidence here was judged to be of very low certainty due to the open-label design of all included studies, imprecise effect estimates with wide CIs, and a limited number of trials (only two contributed to the MACEs analysis). Our findings align with the conclusions from a meta-analysis in 2016, which demonstrated that while IV iron was more effective in raising hemoglobin levels, there was no clear signal of harm in terms of mortality or serious adverse events [13]. Nonetheless, the generalizability of this conclusion remains limited by heterogeneity in oral iron formulations, treatment durations, and the small scale of available RCTs in the dialysis population. These limitations highlight a critical gap in the literature, where adequately powered, blinded trials comparing IV to oral iron on hard clinical endpoints are still lacking. To improve the certainty of evidence in this area, future RCTs should aim to use double-blind designs to reduce performance and detection bias, recruit larger patient populations to enhance statistical power, and incorporate longer follow-up durations to capture clinically meaningful events. Standardized outcome definitions and stratification by baseline iron status would further strengthen the applicability and interpretability of findings.

Our most notable finding emerged from the analysis of high-dose vs low-dose IV iron. Pooled data from six studies indicated that high-dose regimens were associated with a statistically significant reduction in all-cause mortality, without evidence of increased cardiovascular risk. This result was largely driven by the large PIVOTAL trial [32], which has previously shown that proactive, higher-dose IV iron was superior to reactive, lower-dose strategies in reducing cardiovascular events and death. However, our sensitivity analysis revealed that this association lost statistical significance after excluding the PIVOTAL study, suggesting that the overall estimate may be heavily influenced by a single trial. Although our findings are in line with recent analyses such as Zhang et al. [46], which also reported improved hematologic parameters and reduced ESA use with high-dose iron, the low number of events and variation in dosing thresholds between studies still warrant cautious interpretation. The GRADE assessment rated this evidence as moderate certainty, recognizing the consistent direction of effect but noting the potential for residual confounding and limited generalizability. Although our analysis indicated a potential mortality benefit with high-dose IV iron, we were unable to explore whether this benefit varied by baseline iron status due to inconsistent reporting of subgroup-specific outcomes across trials. Most included studies used different ferritin and TSAT thresholds and did not provide stratified results. Future studies should evaluate whether patient subgroups defined by iron indices derive differential prognostic benefit from higher iron dosing strategies.

This study has several strengths. We conducted a comprehensive literature search across six databases to capture a broad range of studies relevant to the dialysis population. All included studies were RCTs, minimizing bias compared to prior meta-analyses that included observational data. We also stratified analyses by key clinical comparisons to provide a more granular understanding of IV iron’s prognostic impact. Risk of bias and evidence certainty were systematically assessed using Cochrane and GRADE methodologies, and sensitivity analyses confirmed the robustness of our findings across comparisons.

Nonetheless, several limitations should be acknowledged. First, the number of eligible RCTs for each comparison was small (2–6 per outcome), which limits the power to detect modest but clinically meaningful differences. This also precluded formal testing for publication bias in some analyses, although funnel plots appeared symmetrical. Second, most included studies were open-label, particularly in the IV vs oral iron comparison, introducing the possibility of performance and detection bias. Third, there was considerable heterogeneity in iron formulations, doses, and administration schedules, making direct comparisons between studies challenging. In particular, the considerable variability in IV iron dosing strategies, formulations, and administration schedules among the included studies limited our ability to explore dose-response relationships in depth. This limitation is compounded by differences in baseline iron status and the lack of patient-level data, which precluded more granular subgroup analyses. Future studies should aim to standardize iron dosing protocols or provide individual-level data to better elucidate how dosing variation may influence clinical outcomes. Fourth, few trials explicitly reported cardiovascular outcomes as primary endpoints, and definitions of MACEs varied. Finally, the relatively short follow-up durations limit our ability to assess long-term safety.

Our findings have several clinical implications. First, IV iron therapy appears safe when used in contemporary hemodialysis care and does not confer an increased risk of mortality or cardiovascular events compared to placebo or oral iron. This supports current guideline recommendations favoring IV iron as the preferred repletion strategy in dialysis patients with iron deficiency [47]. Second, high-dose IV iron may offer additional survival benefits, especially when implemented proactively and with appropriate monitoring, as demonstrated in the PIVOTAL trial [48]. However, in the absence of broader confirmatory evidence, clinicians should consider individual patient risk factors, including infection risk and iron overload, when tailoring iron therapy [9]. Third, the lack of clear benefit from IV over oral iron in reducing hard outcomes underscores the need for further large-scale trials to explore whether differences in iron delivery routes translate into prognostic gains in different CKD subpopulations.

## Conclusion

In conclusion, this meta-analysis suggests that IV iron therapy in patients on maintenance hemodialysis does not significantly affect mortality or cardiovascular outcomes when compared to placebo or oral iron. High-dose IV iron may reduce mortality compared to low-dose regimens; however, this finding requires cautious interpretation given its dependence on a single large trial. Future well-powered, blinded RCTs with long-term follow-up are needed to validate these findings and better define the optimal dosing strategies for IV iron in dialysis patients.

## Supplemental data

**Detailed search strategy for each database:**

**PubMed**

(“Iron” [Mesh] OR “Iron Compounds” [Mesh] OR “iron repletion” OR “intravenous iron” OR “ferric carboxymaltose” OR “ferric derisomaltose” OR “iron isomaltoside 1000” OR “iron sucrose” OR “iron supplementation” OR “iron therapy” OR “ferumoxytol”) AND (“Renal Dialysis” [Mesh] OR “dialysis” OR “hemodialysis”) AND (“Randomized Controlled Trial” [Publication Type] OR “random” [tiab] OR “randomized” [tiab] OR “randomised” [tiab] OR “randomly” [tiab] OR “controlled” [tiab] OR “placebo” [tiab]) AND (“Mortality” [Mesh] OR “Cardiovascular Diseases” [Mesh] OR “mortality” OR “death” OR “deaths” OR “adverse events” OR “survival” OR “cardiac” OR “heart” OR “cardiovascular” OR “prognosis”)

**Embase**

(“iron repletion”/exp OR “intravenous iron”/exp OR “ferric carboxymaltose” OR “ferric derisomaltose” OR “iron isomaltoside 1000” OR “iron sucrose” OR “iron supplementation” OR “iron therapy” OR ”ferumoxytol”) AND

(“hemodialysis”/exp OR “dialysis”) AND (“randomized controlled trial”/exp OR “random”:ab,ti OR “randomized”:ab,ti OR “randomised”:ab,ti OR “randomly”:ab,ti OR “control”:ab,ti OR “placebo”:ab,ti) AND (“mortality”/exp OR “death”:ab,ti OR “deaths”:ab,ti OR “cardiovascular disease”/exp OR “heart”:ab,ti OR “cardiac”:ab,ti OR “survival”:ab,ti OR “prognosis”:ab,ti OR “adverse event”:ab,ti)

**Cochrane Library**

(“iron repletion” OR “intravenous iron” OR “ferric carboxymaltose” OR “ferric derisomaltose” OR “iron isomaltoside 1000” OR “iron sucrose” OR “iron supplementation” OR “iron therapy” OR “ferumoxytol”) AND (“dialysis” OR “hemodialysis”) AND (“randomized” OR “randomised” OR “randomly” OR “control” OR “placebo”) AND (“mortality” OR “death” OR “deaths” OR “adverse events” OR “survival” OR “cardiac” OR “heart” OR “cardiovascular” OR “prognosis”)

**Web of Science**

TS═(“iron repletion” OR “intravenous iron” OR “ferric carboxymaltose” OR “ferric derisomaltose” OR “iron isomaltoside 1000” OR “iron sucrose” OR “iron supplementation” OR “iron therapy” OR “ferumoxytol”) AND TS═(“dialysis” OR “hemodialysis”) AND TS═(“random” OR “randomized” OR “randomised” OR “randomly” OR “control” OR “placebo”) AND TS═(“mortality” OR “death” OR “deaths” OR “adverse events” OR “survival” OR “cardiac” OR “heart” OR “cardiovascular” OR “prognosis”)

**Wanfang**

(“
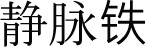
” OR “
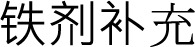
” OR “
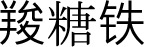
” OR “
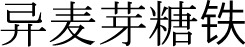
” OR “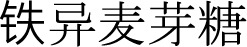 1000” OR “
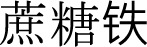
” OR “
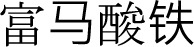
” OR “
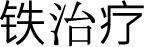
”) AND (“
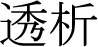
” OR “
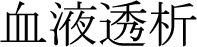
”) AND

(“
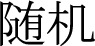
” OR “
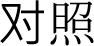
” OR “
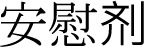
”) AND (“
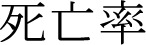
” OR “
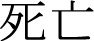
” OR “
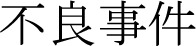
” OR “
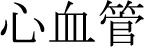
” OR “
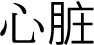
” OR “
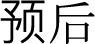
” OR “
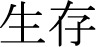
”)

**China National Knowledge Infrastructure (CNKI)**

(“
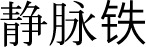
” OR “
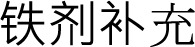
” OR “
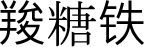
” OR “
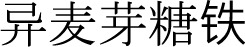
” OR “
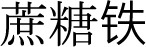
” OR “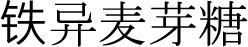 1000” OR “
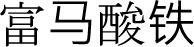
” OR “
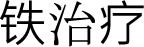
”) AND (“
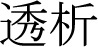
” OR “
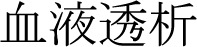
”) AND

(“
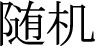
” OR “
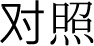
” OR “
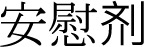
”) AND (“
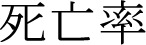
” OR “
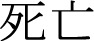
” OR “
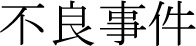
” OR “
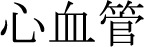
” OR “
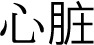
” OR “
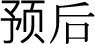
” OR “
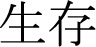
”)

## Data Availability

All data generated or analyzed during this study are included in this published article.
